# Modeling the Cost-Effectiveness of Health Care Systems for Alcohol Use Disorders: How Implementation of eHealth Interventions Improves Cost-Effectiveness

**DOI:** 10.2196/jmir.1694

**Published:** 2011-08-11

**Authors:** Filip Smit, Joran Lokkerbol, Heleen Riper, Maria Cristina Majo, Brigitte Boon, Matthijs Blankers

**Affiliations:** ^1^Trimbos Institute (Netherlands Institute of Mental Health and Addiction)Centre of Prevention and Brief InterventionUtrechtNetherlands; ^2^VU University Medical CentreDepartment of Epidemiology and BiostatisticsEMGO+ Institute for Health and Health Care ResearchAmsterdamNetherlands; ^3^University of AmsterdamDepartment of Business and Industrial StatisticsAmsterdamNetherlands; ^4^VU UniversityDepartment of Clinical PsychologyAmsterdamNetherlands; ^5^GGZinGeestRegional Mental Health Service CentreVU University Medical CentreAmsterdamNetherlands; ^6^Tor Vergata UniversityDepartment of Financial Economics and Quantitative MethodsRomeItaly; ^7^Trimbos Institute (Netherlands Institute of Mental Health and Addiction)Centre of Brief Intervention and PreventionUtrechtNetherlands; ^8^Amsterdam Institute for Addiction ResearchAcademic Medical CentreUniversity of AmsterdamAmsterdamNetherlands

**Keywords:** Alcohol-related disorders, early intervention, health care systems, cost-effectiveness

## Abstract

**Background:**

Informing policy decisions about the cost-effectiveness of health care systems (ie, packages of clinical interventions) is probably best done using a modeling approach. To this end, an alcohol model (ALCMOD) was developed.

**Objective:**

The aim of ALCMOD is to estimate the cost-effectiveness of competing health care systems in curbing alcohol use at the national level. This is illustrated for scenarios where new eHealth technologies for alcohol use disorders are introduced in the Dutch health care system.

**Method:**

ALCMOD assesses short-term (12-month) incremental cost-effectiveness in terms of reductions in disease burden, that is, disability adjusted life years (DALYs) and health care budget impacts.

**Results:**

Introduction of new eHealth technologies would substantially increase the cost-effectiveness of the Dutch health care system for alcohol use disorders: every euro spent under the current system returns a value of about the same size (€ 1.08, ie, a “surplus” of 8 euro cents) while the new health care system offers much better returns on investment, that is, every euro spent generates € 1.62 in health-related value.

**Conclusion:**

Based on the best available evidence, ALCMOD's computations suggest that implementation of new eHealth technologies would make the Dutch health care system more cost-effective. This type of information may help (1) to identify opportunities for system innovation, (2) to set agendas for further research, and (3) to inform policy decisions about resource allocation.

## Introduction

Alcohol use disorders are a leading cause of disease burden [1,2] and are associated with substantial economic costs [3-5]. Therefore, curbing alcohol use has long been recognized as an important public health objective [6,7]. Health care systems play a crucial role in achieving this objective, but most health care systems offer room for improvement in terms of greater efficiency. This begs the question what type of health care system (ie, what mix of interventions) is optimal. We could provisionally define an optimal health care system in terms of meeting the following criteria: the health care system needs to be acceptable to its recipients, scalable to absorb increasing demands for health care, effective to generate the required health gains, and economically affordable to become sustainable over time. Public health planners need ways to design health care systems that optimize these criteria, compare the relative advantage of newly designed systems with the current one, and choose the most cost-effective system. This is a daunting but important task.

However, this task might be facilitated with a simulation model, which can compare a “base-case” scenario (eg, the current mix of clinical interventions) with an alternative (hypothetical) scenario consisting of new interventions or a different mix of interventions. In order for it to be helpful, the model should be able to evaluate the relative advantage of one system over another in terms of incremental cost-effectiveness and be used as an aid to decision-making.

With these aims in mind, we developed an alcohol model (ALCMOD) that can address the above issues. Developing ALCMOD was conducted within the framework of the World Health Organization's International Action Plan on Implementing eHealth Technologies for Substance Abuse. In this context, we wanted to shed light on the population-level cost-effectiveness of health care systems for alcohol use disorders before and after the introduction of new eHealth technologies in Belarus, Brazil, India, Mexico, and the Netherlands. ALCMOD is programmed in Microsoft Excel 2007, because Excel is available on most computers.

The purpose of this paper is to describe ALCMOD's input and output and to take an in-depth look at the model's throughput: its computational strategies, the underlying assumptions, and its limitations. One such limitation is ALCMOD's focus on short-term impacts. Restricting the time horizon to 1 year was a conscious choice because there are several alcohol use disorders (heavy, hazardous, and harmful use and alcohol dependence; see Textbox 1 for definitions) and a lack of empirical data that help to quantify the longer-term treatment effects and relapse rates for each of the alcohol use disorders. By contrast, simulation of short-term health and budget impacts is straightforward and requires fewer assumptions. Strengths of ALCMOD include its ability to evaluate combinations of interventions, its adaptability to different populations and settings, its capacity to handle uncertainty in input parameters, and the way it incorporates coverage and adherence rates for each of the modeled interventions.

We illustrate ALCMOD's computations for the base-case of usual care in the Netherlands versus an alternative scenario consisting of usual care augmented with three eHealth interventions: the DrinkTest, DrinkingLess, and an online therapist-led treatment for problem drinking, termed OnlineTreatment henceforth. The DrinkTest is a brief online intervention consisting of screening one's alcohol use followed by automated personalized advice. DrinkingLess is an online four-step cognitive behavioral intervention. The steps in DrinkingLess are: (1) exploring one's alcohol use, (2) goal setting, (3) behavioral change, and (4) maintenance. Both the DrinkTest and DrinkingLess have been evaluated in randomized trials and meta-analytically and were found to be effective in curbing alcohol use [9-12]. Both the DrinkTest and DrinkingLess are pure self-help interventions, but OnlineTreatment is a therapist-led intervention. Communication between participant and therapist is conducted over the Internet in seven synchronous written chat sessions of 45 minutes each. The sessions are thematically structured and cover themes such as goal setting, self-control techniques, monitoring, recognizing situations that incur a risk of relapse, and relapse prevention techniques.

**Alcohol use disorders**

Alcohol use disorders from the lexicon of alcohol and drug terms published by the World Health Organization [8]

- Abstinence is defined as refraining from drinking alcoholic beverages.
- Moderate drinking is the consumption of alcohol that does not exceed guidelines for moderate drinking in terms of volume or quantity per occasion.
- Heavy drinking is defined as drinking in excess of the standard of moderate drinking (see moderate drinking, above).
- Hazardous use (*International Classification of Disease, Tenth Revision* [*ICD-10*] code Z72.1) is a pattern of heavy drinking and/or binge drinking that carries with it a risk of harmful consequences to the drinker. These consequences may be detrimental to physical or mental health or have adverse social consequences to the drinker or others. Other potential consequences include worsening of existing medical conditions or psychiatric illnesses, injuries caused to self or others due to impaired judgment after drinking, high-risk sexual behaviors while intoxicated, and worsening of personal or social interactions.
- Harmful drinking (*ICD-10* code F10.1) is a pattern of drinking that is causing damage to health. The damage may be either physical (eg, liver cirrhosis from chronic drinking) or mental (eg, depressive episodes secondary to drinking). Harmful patterns of use are often criticized by others and are sometimes associated with adverse social consequences of various kinds. Harmful drinking has persisted for at least 1 month or has occurred repeatedly over the past 12-month period; subject does not meet criteria for alcohol dependence.
- Alcohol dependence (*ICD-10* code F10.2) At least 3 of the following criteria are met: tolerance; withdrawal symptoms; impaired control; preoccupation with acquisition and/or use; persistent desire or unsuccessful efforts to quit; sustains social, occupational, or recreational disability; and use continues despite adverse consequences.

OnlineTreatment has been evaluated in a randomized trial [13]. Preliminary results (not yet published) indicate that OnlineTreatment is effective and cost-effective. It is worth noting that the three eHealth interventions increase in intensity and could be used in a stepped-care framework, thus starting with the least intensive intervention, the DrinkTest, and moving up to the more intensive levels of DrinkingLess and OnlineTreatment, if so required.

The emergence of evidence-based eHealth technologies offers opportunities for innovation in existing health care systems. The new technologies may help to reach population segments that were hitherto not reached because they live in hard to reach rural areas or because they may have shied away from face-to-face delivered health services out of fear of stigma. The new technologies are also very scalable, thus allowing people to access health care services in an unprecedented way. In addition, the new eHealth technologies could be cost-effective, especially when offered as well-structured self-help interventions or as interventions with (minimal) therapist support. Considering the global health gap with regard to the alcohol use disorders [6], these developments could become quite important. However, to date there is only limited evidence for the cost-effectiveness of eHealth interventions [14]. For these reasons, it is opportune to conduct a population-level health-economic evaluation of the possible health gains and budget impacts of adding new eHealth technologies to the existing health care system for alcohol use disorders.

## Methods

### Target Population

By way of input, ALCMOD requires data that describe key characteristics of the target population. Selecting the name of the country will automatically trigger ALCMOD to upload the age and gender distribution of the population of the selected country and the corresponding mortality rates. ALCMOD also needs to know the size of the target population, and in the Netherlands, the target population consists of 993,200 men and 222,800 women aged 18 to 69 years who could be classified as *problem drinkers* [15]. Other required input is the preintervention profile of the target population based on the Alcohol Use Disorders Identification Test, the AUDIT [16-19]. The decision to base ALCMOD on the AUDIT was motivated by the idea that the AUDIT is globally used. Moreover, new eHealth interventions will screen participants with the AUDIT. Thus, even when a country has no AUDIT data yet, these data are likely to become available via eHealth interventions in the near future. In the Netherlands, data from the AUDIT are available and can be automatically uploaded in ALCMOD.

### Intervention Packages

ALCMOD allows a description of the intervention mix representing the base-case scenario and the designing of an alternative scenario with a different mix of interventions or new interventions added to existing ones. In ALCMOD's default setting, a range of interventions—both face-to-face interventions and eHealth interventions—are shown for heavy, hazardous, and harmful alcohol use and alcohol dependence. Two parameters need to be set for each of the interventions: the coverage rate and the adherence rate.

#### Coverage Rate

When some of the interventions shown in ALCMOD's default setting are not available in a country, then their coverage rate has to be set to 0%. This is equivalent to saying that the intervention is not offered in a country. Other interventions might be available for every person belonging to the target population, and the coverage rate is then set to 100% (universal coverage). However, due to the many obstacles to full implementation, the coverage rate of most interventions is somewhere between 0 and 100% and can be set accordingly in ALCMOD.

#### Adherence Rate

Recipients of interventions might be less than willing or unable to fully comply with the intervention, and the degree of adherence is likely to moderate treatment response. Therefore, the adherence rate is an important parameter when evaluating the effectiveness of interventions. Adherence rates may be obtained from the literature, experts, or via focus groups in the target population.

The idea is that health care scenarios can be developed by changing the level of coverage for a series of interventions. Table 1 shows the settings for the three scenarios that we modeled: (1) the current Dutch health care system for alcohol use disorders without eHealth interventions (base-case scenario), (2) the Dutch health care system augmented with the eHealth interventions (alternative scenario 1), and (3) the Dutch health care system where face-to-face interventions have been substituted for 50% by the new eHealth interventions (alternative scenario 2).

**Table 1:** Modeled scenarios: coverage rates (%) for each of the interventions

Target Group Alcohol Use Disorder	Intervention	Base-Case Scenario	Alternative Scenario 1	Alternative Scenario 2
				
Heavy	Brief face-to-face intervention^a^	10	10	5
	Online brief intervention ^b^	0	5	5
				
Hazardous	Brief face-to-face intervention^a^	10	10	5
	Online brief intervention^b^	0	5	5
	Behavioral intervention^c^	6	6	3
	Online behavioral intervention^d^	0	6	3
				
Harmful	Behavioral intervention^c^	9	9	3
	Online behavioral intervention^d^	0	9	3
	Online therapist-led treatment^e^	0	9	3
	Detox and acamprosate^f^	5	5	5
	Aftercare and rehab with AA^g^	5	5	5
				
Dependence	Behavioral intervention^c^	5	5	2.5
	Online therapist-led treatment^e^	0	5	2.5
	Detox and acamprosate^f^	5	5	5
	Aftercare and rehab with AA^g^	5	5	5
				

^a^ Brief face-to-face is modeled as a brief intervention consisting of screening followed by personalized feedback by a physician usually in a single session (< 10 minutes), occasionally in two sessions (one for screening, the other for personalized feedback).

^b^ Online brief intervention is modeled as online screening and automated personalized feedback (DrinkTest).

^c^ Behavioral intervention is modeled as eight to ten sessions of individual cognitive behavioral therapy (CBT) under the guidance of a therapist, followed by one booster session.

^d^ Online self-help intervention (DrinkingLess) is modeled as four (range 3 to 12) sessions of online interactive CBT-based self-help preceded by referral by a general practitioner (GP).

^e^ Online therapist-led intervention is modeled as eight sessions of online therapist-led CBT.

^f^ Detox is modeled as 1-week ambulatory detoxification followed by clinical management with acamprosate.

^g^ Aftercare and rehabilitation is modeled as participation in Alcoholics Anonymous (AA) over 12 months.

The choice of intervention mix was informed by Room et al and Benegal et al [17,19] and the Dutch multidisciplinary guideline for the treatment of alcohol use disorders [20]. The choice of interventions was also motivated by two additional considerations: availability of evidence of the intervention's effectiveness in the meta-analytical literature [21] and the nonoverlapping *independent* nature of the interventions such that each intervention could be added to other interventions without creating overlap for a specific alcohol use disorder. Finally, the scenarios have been simplified by assuming that all interventions are associated with an adherence rate of 50%. This was done to ensure that differences in the cost-effectiveness ratios are due to fundamental differences in health technologies and not simply an effect of greater or lesser treatment adherence. However, it is possible to adjust adherence rates in ALCMOD. After all, some interventions might be associated with better or poorer adherence, and adherence itself might be amenable to intervention such as motivational enhancement. Changing the adherence parameters allows evaluation of these issues.

### Cost and Effect Parameters

In the ALCMOD default settings, some of the intervention parameters have been preset and need not be changed but can be changed if so required. These parameters are the costs and the effects of the interventions.

#### Costs

ALCMOD's default setting makes use of the full economic cost price of each of the interventions. To be precise, the costs are the per-participant costs of delivering an intervention expressed in euros (€) for the Netherlands in the reference year 2009 (see Table 2). The costs are based on the amount of resources (labor, facilities, and supplies) used for offering the intervention during its postimplementation stage. We made our own costing tool to estimate the costs (in euros) of interventions in a systematic and uniform way that is in agreement with the Dutch guideline for costing health care interventions [22]. For other countries, the per-participant costs of offering an intervention need to be assessed. These assessments can be carried out with the help of an auxiliary costing tool, for example Cost It, available from WHO's CHOICE website. Neither costs nor effects are discounted because ALCMOD takes a short-term (12-month) perspective.

#### Effects

Intervention effects are expressed as the standardized mean difference, also known as Cohen's d. This metric indicates how many standard units (on a scale of standard deviations) the experimental group has improved relative to a control group on a relevant outcome such a change in drinking behavior. The effect size d is often reported in the meta-analytical literature and gives access to a large body of scientific evidence. We extracted effect sizes at 6- or 12-months follow-up for all the interventions from the meta-analytical literature and our own research (see Table 3) and these values were used to populate ALCMOD with its default parameter settings.

**Table 2:** Per-patient intervention costs in 2009 euros (€) within uncertainty range (based on 1000 simulations)

Target Group Alcohol Use Disorder	Intervention	Costs in Euros	Uncertainty Range (Euros)	
			Low	High
Heavy	Brief face-to-face intervention^a^	58	52	75
	Online brief intervention^b^	10	9	10
				
Hazardous	Brief face-to-face intervention^a^	58	52	75
	Online brief intervention^b^	10	9	10
	Behavioral intervention^c^	2024	1702	2550
	Online self-help intervention^d^	207	198	224
				
Harmful	Behavioral intervention^c^	2024	1702	2550
	Online self-help intervention^d^	207	198	224
	Online therapist-led intervention^e^	764	227	1451
	Detox and acamprosate^f^	1800	1620	2232
	Aftercare and rehab with AA^g^	500	250	750
				
Dependence	Behavioral intervention^c^	2024	1702	2550
	Online therapist-led intervention^e^	1276	979	1408
	Detox and acamprosate^f^	1800	1620	2232
	Aftercare and rehab with AA^g^	500	250	750
				

^a^ Brief face-to-face intervention modeled as screening at € 5.70 followed by 1 or 2 (Poisson distributed) 10-minute contacts with GP at € 32.03 per contact

^b^ Online brief intervention (DrinkTest) modeled as 40% of target population (N = 1,255,000) reached with information about the website, 8% responding to AUDIT screener and receiving automated personalized feedback. Per-participant annual costs include website upgrading at € 50,000 research at € 50,000 and hosting at € 25,000.

^c^ Behavioral intervention is modeled as 8 to 14 (Poisson distributed) sessions of cognitive behavioral therapy (CBT) under guidance by a therapist, including referral, intake, and one booster session

^d^ Online self-help intervention (DrinkingLess) is modeled as 15% of target population (N = 1,255,000) reached with information about the website 5% uptake rate, and 4 sessions (range 3 to 12) of online CBT-based self-help preceded by referral by a GP. Per-participant annual costs include € 75,000 for website upgrading, € 50,000 for research, € 25,000 for hosting, plus € 75,000 for moderating forum and technical assistance.

^e^ Online therapist-led intervention is modeled as an average of 4 sessions (range 1 to 9) of 45 minutes each of online therapist-led CBT, preceded by GP referral. Per-participant costs include per annum costs of € 8000 for website upgrading, € 5000 for hosting, plus € 2000 for technical assistance.

^f^ Detox is modeled as a 1-week ambulatory detoxification followed by clinical management with acamprosate under the supervision of a substance use disorder treatment specialist and a physician over 3 months.

^g^ Aftercare and rehabilitation is modeled as participation in Alcoholics Anonymous at an average of € 500 (range € 250 to € 750) per patient for a year.

It is worth noting that ALCMOD uses two types of effects: the standardized mean difference, d, which was just discussed, and the impact of an intervention in terms of the percent reduction of pure alcohol intake in grams per day (g/day). The former effect (d) impacts on health-related quality of life (QOL) via changes in disorder severity. ALCMOD uses the percent reduction of pure alcohol intake to model treatment effects on mortality (see below for details). Although ALCMOD can handle different alcohol reduction rates for each of the modeled interventions, we have assumed a pre-post reduction of alcohol intake by 20% for all interventions [21,23], because reduction of alcohol intake was not always reported in the literature. This should not overly distort outcomes because the short-term effects of alcohol use on mortality are small, thus limiting their impact on disease burden as measured by disability-adjusted life years (DALYs). We say this on the understanding that alcohol-related mortality becomes an important, even a dominant, factor when disease burden is modeled out to full life expectancy, especially in the more severe alcohol use disorders.

Here we need to address a final point about the required input for ALCMOD. ALCMOD can be operated in two modes: deterministic and stochastic. In deterministic mode, ALCMOD does not take into account the uncertainty in parameters such as costs and effects. ALCMOD conducts all computations, but only once, and these calculations are primed on the mean value of all parameters. Much of ALCMOD's output, which is based on uncertainty, is then disabled. However, in stochastic mode, ALCMOD can handle uncertainty surrounding the cost (in euros) and effect (d) parameters. Our costing tool assesses the uncertainty in costs with the help of simulations of resource use (with 1000 iterations), and both randomized trials and meta-analyses of trials often report 95% confidence intervals of the effect size d. Thus we assume that costs are surrounded by an uncertainty range, and effects, by a 95% confidence interval, both having a lower and an upper limit. ALCMOD assumes a gamma distribution for costs and the normal distribution for the effect size d. Both distributions can be specified in ALCMOD such that the distributions fit within the lower and upper limits of costs and effects. In stochastic mode, ALCMOD then proceeds with drawing random values from these distributions, conducts all the computations, and repeats this process many times (maximum 10,000 times). This helps to capture uncertainty in the input parameters.

**Table 3:** Effectiveness of the interventions: standardized mean differences, 95% confidence interval for d (95% CI), difference in pure alcohol intake (mg/day) and references

Target Group Alcohol Use Disorder	Intervention	d	95% CI
Heavy	Brief face-to-face intervention^a^	0.26	0.20 to 0.32
	Online brief intervention^b^	0.19	-0.02 to 0.40
Hazardous	Brief face-to-face intervention^c^	0.32	0.23 to 0.42
	Online brief intervention^b^	0.19	-0.02 to 0.40
	Behavioral intervention^d^	0.34	0.12 to 0.56
	Online self-help intervention^e^	0.31	-0.69 to 1.30
Harmful	Behavioral intervention^d^	0.34	0.12 to 0.56
	Online self-help intervention^e^	0.31	-0.69 to 1.30
	Online therapist-led intervention^f^	0.58	0.29 to 0.88
	Detox and acamprosate^g^	0.21	0.14 to 0.29
	Aftercare and rehab with AA^h^	0.28	0.20 to 0.37
Dependence	Behavioral intervention^i^	0.32	0.05 to 0.59
	Online therapist-led intervention^f^	0.59	0.30 to 0.90
	Detox and acamprosate^g^	0.21	0.14 to 0.29
	Aftercare and rehab with AA^h^	0.28	0.20 to 0.37

^a^ Moyer et al's [21] meta-analysis of brief face-to-face interventions in approximately 4300 users meeting criteria of at least heavy drinking.

^b^ Randomized trial of 450 participants presenting with either excessive alcohol consumption (> 20 units weekly) and/or binge drinking (> 5 units on a single occasion on least one day per week) in the past 6 months [9].

^c^ Reanalysis of Beich et al’s [24] meta-analysis of brief face- to-face interventions in 2989 users meeting criteria of hazardous drinking.

^d^ Walters’ [25] meta-analysis based on approximately 320 harmful users.

^e^ Randomized trial of 261 excessive drinkers from the general population [11] where odds ratio (OR) converted into d using Chinn's equation [26].

^f^ Randomized trial of 250 adults with mean AUDIT score of 20 at baseline with intervention was online treatment versus waiting list at 3 months and the AUDIT as outcome [13].

^g^ Mann et al's [27] meta-analysis of 1670 people receiving acamprosate after detoxification where odds ratios converted into d using Chinn's method [26].

^h^ Tonigan et al's [28] meta-analysis of 2097 harmful and dependent users where effect size r converted into d.

^i^ Walters’ [25] meta-analysis based on approximately 210 dependent users.

### ALCMOD's Throughput

#### Differences in Costs

Modeling cost differences between two health care systems is straightforward once the per-participant costs of delivering all modeled interventions have been estimated and when the coverage rates of the interventions have been established. The number of people in the target group (stratified by alcohol use disorder) is then multiplied by the coverage rate of each intervention and multiplied by the appropriate per-participant full economic cost price. The cost analyses are always conducted for both the base-case and alternative scenario, such that the cost difference between two modeled health care systems can be computed and expressed as incremental costs.

#### Differences in Disease Burden

The disability adjusted life year (DALY) is a measure of disease burden in a population. It combines two components of disease burden: morbidity and mortality. The first is related to lesser quality of life due to disability. Mortality arises when illness is associated with premature death. Thus, a DALY can be computed as the sum of years lost due to disability (YLD) plus years of life lost (YLL) due to mortality, hence, DALY = YLD + YLL.

The first term in the DALY equation, YLD, can be computed as the number of cases manifesting with an alcohol use disorder, N (point prevalence), weighted by a disability weight, DW. Thus, YLD = N × DW. DWs range from 0 to 1, where 0 is *no burden* (good health) and 1 refers to a health condition as undesirable as death. Although the literature offers advice for choosing DWs for the alcohol use disorders [29-33], ALCMOD makes no use of DWs that are directly associated with each of the disorder-specific health states. Instead, it computes the (downward) shift in DW as a consequence of the treatment effect d. As said, d is the standardized mean difference indicating how many standard units the treatment group has moved away from the group that received no care. Thus, d is essentially a “health improvement shift” due to intervention. The task at hand, then, is to “translate” the health improvement shift (of size d) into a corresponding shift in DW. This strategy has been developed by Sanderson et al [34] who used a panel of experts for obtaining a conversion factor of 0.18 (95% CI 0.16-0.20) to translate a shift in d into a shift in DW in alcohol use disorders. The change in DW is then multiplied by the appropriate number of people to arrive at an estimate of the number of YLD avoided. When running in stochastic mode, ALCMOD automatically conducts extensive uncertainty analyses around Sanderson's conversion factor.

The second term in the DALY equation, YLL, is calculated as the difference in life expectancy when people reduce drinking levels. We obtained estimates of the gender-specific relative risk, RR, of all-cause mortality attributable to pure ethanol intake (in g/day) using the expression [35], ln(RR) = b_1_*(ln(x+1)) + b_2_*ln(x) + e, where x = grams of pure ethanol intake per day and b_1_ and b_2_ are -0.1030 and 0.0035 for men and -0.0645 and 0.0029 for women, respectively (our own estimates from Gmel's paper [35]). Exponentiating ln(RR) gives the relative risk, RR, and the RRs are then combined with the gender and age-specific mortality rates of the country for which the outcomes are modeled. This produces estimates of changes in life expectancy due to changes in alcohol intake. Because ALCMOD takes a short-term perspective, treatment induced impacts on life expectancy were calculated as the number of avoided deaths in the present year.

The difference in YLD and YLL between the scenarios determines the difference in the disease burden as measured by DALY between two modeled health systems, the so-called incremental effects.

ALCMOD offers the use of a (downward) *attenuation factor* that reduces the carry-over effects from lesser drinking to lesser mortality and better health-related quality of life. After all, it can be assumed that former drinkers still have a higher risk of dying and poorer quality of life than people who never drank before or have been consistent moderate drinkers [36,37]. In other words, returning to less risky drinking levels is assumed to be beneficial but not as beneficial as a history of no drinking or moderate drinking. Hence this attenuation factor, which can be used to conduct sensitivity analyses for further evaluation of this issue. In all subsequent analyses we used a downward adjustment of 20% to be on the conservative side.

#### Combining Costs and Effects

Once the treatment costs and the reductions in DALY disease burden have been computed for each scenario, then it is a small step to also compute the difference between the costs of both health care systems as Δ(C) = C_1_ - C_0_ and the difference between the effects as Δ(E) = E_1_ - E_0_. The ratio, Δ(C)/Δ(E), is the incremental cost-effectiveness ratio (ICER), which tells us if the alternative health care system offers better value for money than the current health care system. 

#### Handling Uncertainty

As indicated, in stochastic mode, ALCMOD takes parameter uncertainty into account. The uncertainty is captured by drawing values from the cost and effect distributions of all interventions in a random way and basing the calculations on these randomly drawn values. This can be repeated n times (in practice 500 times appears to be sufficient) and the outcomes of each of the iterations is stored in vectors of size n of the costs and effects of each of the scenarios, their differences, and the ICER. Following standard health economic modeling routines [37], the vectors are then used to produce ALCMOD's output, such as the mean and the median of the outcomes and several ICER plots and graphs.

## Results

### Preintervention Target Group

In November 2009 we obtained data from the DrinkingLess monitoring system on 1083 women and 2538 men who participated in DrinkingLess. Their mean age was 44.7 years (SD 10.7). Table 4 presents the observed AUDIT distribution for this population and the relative risk (RR) of premature death due to alcohol, as computed by ALCMOD.

**Table 4:** Preintervention characteristics of the target population

AUDIT	Tentative	Men, %	Women, %	RR (Death)	
Score	Label	(n = 987,000)	(n = 267,000)	Men	Women
0 - 1	Abstinent	0.1	0.1	1.00	1.00
2 - 7	Moderate	1.6	3.6	0.86	0.96
8 - 15	Heavy	18.4	23.5	0.95	0.99
16 - 19	Hazardous	22.2	23.5	0.99	1.05
20 - 29	Harmful	50.1	43.4	1.10	1.12
30 - 40	Dependence	7.6	5.9	1.36	1.28

### Comparing Current Care With New eHealth Interventions Added

We begin by comparing the current health care system (base-case scenario) with an alternative scenario where eHealth interventions are added to conventional care. In this comparison, it was assumed that the new eHealth interventions would attract a different segment from the target population—a segment that would otherwise not have been the recipient of conventional care. Making this (unrealistic) assumption is a conscious choice, and we will return to it in the “Discussion” section. The results are as follows.

**Figure 1:**
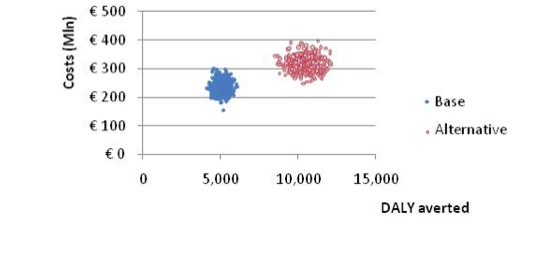
Total costs and effects in millions of euros (base-case scenario versus eHealth interventions added)

The total health care costs in the base-case scenario are € 233 million. Adding new eHealth interventions would raise the health care expenditure to € 319 million, an increase of € 86 million. Under the base-case scenario, 5022 DALYs are averted; under the new scenario, this is doubled to 10,319 averted DALYs, an additional 5296 averted DALYs (including 32 alcohol-related deaths that are avoided under the new scenario). Thus, the alternative health care system delivers more population health albeit at higher costs. Figure 1 provides a corresponding visualization: the scatter of simulated ICERs (due to uncertainty in the input parameters) corresponding to the alternative scenario is placed more to the north (more costs) and more to the east (more health) than the scatter belonging to the current health care system. Now, investing € 86 million for averting 5296 extra DALYs (ie, € 16,053/DALY) raises the question whether that would be money spent wisely. In the Netherlands, the willingness to pay for one averted DALY is about € 80,000 with a lower bound of € 50,000. An even more conservative willingness-to-pay ceiling is customarily set at € 20,000/DALY for nonfatal and mild disorders. It follows that the estimated € 16,053/DALY falls well below any of the usual willingness-to-pay ceilings.

**Figure 2:**
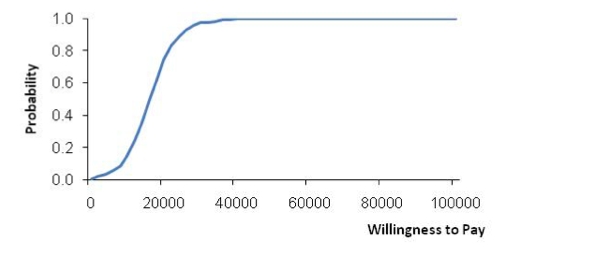
ICER acceptability curve (base-case scenario versus eHealth interventions added)

Figure 2, the ICER acceptability curve, represents a slightly different approach to the same issue. It depicts the probability that we must conclude that the new health care system is more cost-effective than the current system (vertical axis) for a range of willingness-to-pay ceilings (horizontal axis). For the simulated data, Figure 2 shows that the likelihood that the new health care system must be regarded as cost-effective increases sharply with increasing willingness-to-pay ceilings: the probability equals 0% when the willingness to pay for an additional health gain of one DALY averted is € 0, increases to 50% at € 16,000 and to 75% at € 20,000. Beyond € 30,000 the probability approaches certainty, and the conclusion that we must regard the new system as more cost-effective is no longer affected by higher willingness–to-pay levels. Again, accepting the threshold of € 20,000/DALY implies that the new health system compares favorably with the current system in terms of cost-effectiveness.

**Figure 3:**
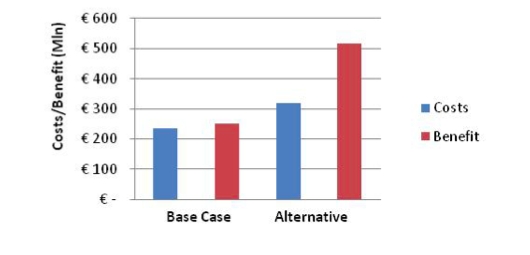
Cost-benefit chart in millions of euros (base-case scenario versus eHealth interventions added)

Assuming for a moment that the willingness to pay for averting one DALY is €50,000, then we could directly compare the costs of the health care system (in euros) with health gains (also expressed in euros) by multiplying the averted DALYs by €50,000. Figure 3, a cost/benefit chart, shows ALCMOD's simulation results. The chart shows that costs and benefits are just balanced under the current health care system, while the benefits clearly outweigh the costs under the new system. To be more precise, every euro spent under the current system returns a value of about the same size (€1.08, ie, a “surplus” of 8 euro cents), while the new health care system offers much better returns on investment: every euro spent generates €1.62 in health-related value.

To summarize, the new health care system, with eHealth interventions added, is associated with higher health care delivery costs overall, but it would be a health care system which is more efficient than the current one, offering better value for money.

### Comparing Current Care With New eHealth Interventions With Partial Substitution

We also simulated another comparison, this one between a base-case scenario that represents the current system and an alternative scenario with eHealth interventions added. In this case, however, the conventional face-to-face interventions are partly substituted by the new eHealth interventions. In this scenario, the coverage rate remains the same before and after the introduction of the eHealth interventions. Such a situation would arise if the eHealth interventions were to tap into the same target population seeking professional help, whether face-to-face, eHealth, or otherwise. In this scenario, the number of people who receive health care remains the same before and after the introduction of the new health technologies, and interventions are competing for the same target population and therefore partially substitute each other.

**Figure 4:**
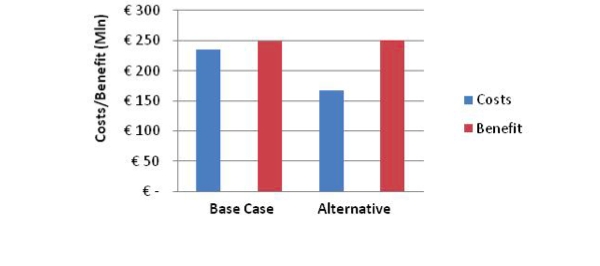
Cost/benefit chart in millions of euros (base-case scenario versus partial substiution scenario)

In this scenario, ALCMOD computes that the number of DALYs averted under both systems is virtually the same: 4984 DALYs under the current system and (exactly) 5000 DALYs under the new system. In other words, partial substitution of conventional face-to-face interventions by eHealth interventions does not have any appreciable impact on population health. However, the overall cost of the new system is much lower at €166 million than the cost of the current system of €234 million, resulting in a cost saving of € 68 million. Figure 4 relays the same information. Again assuming a willingness to pay of €50,000/DALY, the cost-benefit ratio indicates that for every euro invested the generated health revenues are worth €1.06 (ie, 6 euro cents surplus for every euro invested) under the current health care system. This improves to 52 euro cents surplus for every euro invested under the new scenario where the face-to-face interventions have been partly substituted by new eHealth interventions.

## Discussion

### Main Findings

The main rationale for introducing eHealth technologies is to increase timely access to health services, to reduce the costs of delivering health care, and to make more efficient use of the health care workforce. Indeed, ALCMOD's simulation results suggest that widespread implementation of eHealth interventions for alcohol use disorders would help to substantially increase population health in the Netherlands, albeit at higher system costs, when eHealth interventions are added to the existing health care system and more people become the recipients of the expanded system. The cost-effectiveness of the Dutch health system would also substantially improve if the new eHealth interventions were partially replacing some of the current face-to-face interventions. Then, adding eHealth interventions becomes a cost-effective option, because it will produce the same level of population health for a significantly smaller health care budget. The “truth” might be found somewhere between both extremes, because it is unlikely that the new eHealth interventions will exclusively recruit people that would otherwise not have been the recipients of conventional health care (as assumed in the first comparison), while it is also unlikely that the new eHealth interventions will tap into exactly the same pool of health care users (as assumed in second comparison). At any rate, both extreme scenarios carry the message that widespread introduction of eHealth technologies would help to substantially increase the efficiency of the Dutch health care system overall, with a more favorable cost-benefit ratio either way.

### Strengths and Limitations of ALCMOD

One of the benefits of a simulation model is that it helps to organize vast fields of knowledge across several disciplines. In the case of ALCMOD, these disciplines encompass addiction epidemiology and health economics, while the evidence that supports effect parameters is drawn from randomized clinical trials, meta-analyses, and evidence-based clinical guidelines. In addition, a model makes all the necessary information available in a dynamic form, permitting “what if” analyses. This could be of assistance to policy formulation. ALCMOD is therefore best seen as a decision-making support tool, capable of giving almost instant feedback on policy-makers' attempts to find an optimal solution in the context of constrained decision-making in a complex environment. ALCMOD can also be employed for setting research agendas. After all, it helps to identify those parameters that have an impact on health gains and costs. When some of these parameters are surrounded by a nonnegligible degree of uncertainty, then empirical research is recommended, with the aim of reducing uncertainty in those parameters. Furthermore, ALCMOD can assist in identifying opportunities for system innovation by simulating hypothetical interventions, for example, an adjunctive intervention that helps to enhance treatment adherence. Among other strengths of ALCMOD are its adaptability to other countries, settings, and target groups and its capability to explicitly model treatment coverage and adherence rates. Finally, ALCMOD conducts automated multivariate uncertainty analyses to quantify uncertainty in costs, effects, and related parameters.

ALCMOD is subject to several limitations that need to be taken into account when interpreting ALCMOD's outcomes. First, ALCMOD's outcomes are modeled as *steady-state* population averages, and it is not clear when a health care system finds equilibrium after the introduction of new health technologies. This is unlikely to occur instantaneously and might take as long as several years. Second, it should be borne in mind that the introduction of new health technologies entails costs of its own, but the costs of introducing new technologies are not incorporated in ALCMOD's output. In fact, ALCMOD's output captures only the costs of offering a package of interventions once the interventions have been fully implemented. However, it will always take effort, time, and expenditure before the results of an improved health care system become visible in real life. Third, introduction of eHealth technologies may have unforeseen consequences that may increase longer-term health care costs, for example, by supply-induced demand for health care, thus attracting people to the health care system who otherwise would not have become dependent on (expensive, face-to-face delivered) health care. Fourth, it should be understood that ALCMOD focuses on short-term health impacts. Thus, ALCMOD ignores the longer-term impacts on quality of life, mortality, and health care utilization and it should be understood that longer-term impacts depend, in part, on a wide range of alcohol-related disorders that usually occur later in life. Since these longer-term effects are mainly related to the more severe alcohol use disorders, ALCMOD is unlikely to capture the full benefits of interventions for the severe disorders and may thus give undue weight to the less severe disorders. Fifth, ALCMOD is limited in that it only models clinical interventions while disregarding other alcohol-control options, such as banning alcohol advertising, taxing, restricting access to alcoholic beverages, and improving road safety, although these nonclinical interventions are likely to be (very) cost-effective [38]. In the same vein, ALCMOD regards only the cost impacts incurred by the health care system, while disregarding costs and cost-offsets outside the health care system, such as patients' out-of-pocket payments to access services, changes in labor productivity, and costs incurred by the criminal justice system. To summarize ALCMOD's basic assumptions: ALCMOD only models incremental health gains and health care delivery costs over the shorter time horizon, assuming a steady state in the modeled health care systems. See Textbox 2 for a summary of ALCMOD's assumptions and their justifications.

**ALCMOD's assumptions and justifications**

General assumptions:

- ALCMOD disregards the longer-term downstream costs, cost offsets, and health effects due to less drinking because the empirical literature rarely reports treatment effects beyond 12 months.
- Per-participant costs are assumed to follow a gamma distribution [38].
- Treatment effects, expressed in standardized mean difference scores, d, are assumed to follow the standard normal distribution, because d is almost equivalent to a *z*-score.
- The YLD (quality of life) differential is based on Sanderson et al’s conversion factor [34], which translates a change in disorder severity of size d induced by an intervention into a corresponding shift in the disability weight (DW) used in the YLD calculations.
- The YLL (mortality) differential is based on Gmel et al’s [35] relative risk of all-cause mortality stratified for level of pure ethanol intake (g/day).
- Costs and DALY outcomes have not been discounted because the focus is on short-term (<12 months) postimplementation (steady state) health and budget impacts.

Additional assumptions for the current simulations:

- The AUDIT distribution obtained from DrinkingLess is representative for the target population because this is a population of (former) problem drinkers still at risk of an alcohol use disorder and willing to seek treatment.
- Adherence rate is 50% for all interventions because a constant figure would help to obtain a clear view on cost-effectiveness due to fundamental changes in health care technologies.
- Alcohol intake is reduced by 20% after all interventions because the short-term contribution of YLL to the DALY disease burden is virtually negligible.
- All treatment effects on YLD and YLL are attenuated by 20% because the detrimental health effects of problem drinking are likely to linger on—even after return to moderate drinking or abstinence.

### Conclusion

It is not immediately clear if our findings are valid for countries other than the Netherlands. After all, in low-income countries, labor might be less costly than the capital inputs required for the new eHealth technologies. Also the population's access to the Internet could be an issue. Moreover, one could encounter cultural obstacles to using the Internet for alcohol use disorders. Such factors might impinge on coverage and adherence rates and mitigate impacts on population health, ultimately diminishing the cost-effectiveness of new health technologies. To illustrate, in the Netherlands, close to 90% of the population has access to the Internet, and Internet usage is distributed fairly evenly across demographic groups, but in other countries, Internet usage might be concentrated in only some population segments. In addition, it is worth noting that the emergence of mobile technologies may offer an opportunity to offer eHealth interventions for population segments that otherwise might be hard to reach. Therefore, the question as to whether eHealth will deliver the same benefits to other countries is best addressed per country, per setting, and per target group. Ante hoc assessment of the cost-effectiveness of innovations in health care systems may help to inform policy decisions. ALCMOD was created with exactly these aims in mind.

We recommend that ALCMOD be used in an iterative consensus building process that encompasses all pertinent stakeholders (eg, health care users, health care providers, health care financiers, and health policy planners) who can review and make amendments to modeled scenarios. Recently, we had an encouraging experience with such an approach while using a similar model for the treatment of depressive disorder. In any case, we would advise against using ALCMOD as an autopilot for policy making. After all, setting priorities for health care delivery is about acceptability and equity, as well as about cost-effectiveness considerations. As always, we need to base decisions on the best judgments and evidence available, but the evidence that informed ALCMOD points toward the conclusion that eHealth interventions can help to bridge the mental health gap by bringing scalable and cost-effective health services within reach of all who have access to the Internet—literally at their fingertips.

## Abbreviations

AA: Alcoholics Anonymous
ALCMOD: alcohol model
AUDIT: Alcohol Use Disorders Identification Test
CBT: cognitive behavioral therapy
DALY: disability adjusted life year
ICER: incremental cost-effectiveness ratio
DW: disability weight
GP: general practitioner
OR: odds ratio
QOL: quality of life
RR: relative risk
YLD: years lost due to disability
YLL: years of life lost
